# Supplementary material for: Quality of life and health status in older adults (≥65 years) up to five years following colorectal cancer treatment: Findings from the ColoREctal Wellbeing (CREW) cohort study
Source: PLoS One. 2022 Jul 14;17(7):e0270033. doi: 10.1371/journal.pone.0270033 (PMC9282586; doi:10.1371/journal.pone.0270033)
Supplement: S1 Appendix — (DOCX) [file pone.0270033.s001.docx]

**S1 Appendix****. Significant predictors (p<0.05) in the first regression model for each outcome separately by domain.**  Full results are available on request

| **Domains** | **Predictors** | **QLACS-GSS** | **Health Status (EQ-5D) outcomes** | | | | |
| --- | --- | --- | --- | --- | --- | --- | --- |
|  |  |  | **Mobility** | **Self-care** | **Usual activities** | **Pain/ discomfort** | **Anxiety/ depression** |
| **1. Socio-demo** | **Age groups** | Yes | Yes |  | Yes |  |  |
|  | **Gender** |  |  |  | Yes |  | Yes |
|  | **Deprivation quintiles** |  | Yes | Yes | Yes |  |  |
| **2. Environmental** | **Living alone status** |  |  |  |  |  |  |
|  | **MOS-SSS (cut off=100)** | Yes | Yes | Yes | Yes |  | Yes |
| **3. Clinical** | **Tumour site** |  |  |  | Yes | Yes |  |
|  | **Dukes’ Stage** |  |  |  |  |  | Yes |
|  | **Nodal Status** |  |  | Yes | Yes |  |  |
|  | **Comorbidities** | Yes | Yes | Yes | Yes | Yes | Yes |
| **4. Treatment** | **Stoma status** | Yes |  | Yes | Yes | Yes | Yes |
|  | **Adjuvant therapy** |  |  | Yes |  |  |  |
|  | **Neo-adjuvant therapy** |  |  |  |  |  |  |
| **5. Personal** | **LORIG (4 groups)** | Yes | Yes | Yes | Yes | Yes | Yes |
|  | **CES-D (cut off=20)** | Yes |  |  |  | Yes | N/A |
|  | **STA-I (cut off=40)** | Yes |  |  |  |  | N/A |

Note: each model per domain was adjusted for the waves of participation after baseline (at least one wave between 3m and 60m).
