# Supplementary material for: Quality of life and health status in older adults (≥65 years) up to five years following colorectal cancer treatment: Findings from the ColoREctal Wellbeing (CREW) cohort study
Source: PLoS One. 2022 Jul 14;17(7):e0270033. doi: 10.1371/journal.pone.0270033 (PMC9282586; doi:10.1371/journal.pone.0270033)
Supplement: S2 Appendix — (DOCX) [file pone.0270033.s002.docx]

**S2 Appendix.** **Distribution of baseline characteristics for older (>65 years) and younger (<65 years) CREW participants**

| **Baseline characteristics** | | **Aged <65 (n=253)** | | | **Aged 65+ (n=501)** | | **P-value ^1^** |
| --- | --- | --- | --- | --- | --- | --- | --- |
|  |  | *median (LQ, UQ)* / n (%) | | | *median (LQ, UQ)* / n (%) | |  |
| **Outcomes** | ***QLACS-GSS*** | *71.0 (55.0, 91.0)* [n=241] | | | *67.0 (52.0, 82.0)* [n=436] | | *0.022 ** |
|  | **EQ-5D Mobility** |  |  | |  |  |  |
|  | Having no problems | 212 | (83.79) | | 365 | (72.85) | 0.002 ** |
|  | Having problems | 41 | (16.21) | | 129 | (25.75) |  |
|  | **EQ-5D Self-care** |  |  | |  |  |  |
|  | Having no problems | 237 | (93.68) | | 446 | (89.02) | 0.042 * |
|  | Having problems | 16 | (6.32) | | 47 | (9.38) |  |
|  | **EQ-5D Usual activities** |  |  | |  |  |  |
|  | Having no problems | 176 | (69.57) | | 323 | (64.47) | 0.251 |
|  | Having problems | 76 | (30.04) | | 172 | (34.33) |  |
|  | **EQ-5D Pain/discomfort** |  |  | |  |  |  |
|  | Having no problems | 123 | (48.62) | | 239 | (47.70) | 0.283 |
|  | Having problems | 129 | (50.99) | | 253 | (50.50) |  |
|  | **EQ-5D Anxiety/depression** |  |  | |  |  |  |
|  | Having no problems | 145 | (57.31) | | 348 | (69.46) | 0.003 ** |
|  | Having problems | 106 | (41.90) | | 147 | (29.34) |  |
| **Block 1 - socio-demo** | **Gender** |  | |  |  |  |  |
|  | Male | 143 | | (56.52) | 301 | (60.08) | 0.348 |
|  | Female | 110 | | (43.48) | 200 | (39.92) |  |
|  | **Deprivation index** |  | |  |  |  |  |
|  | 1st quintile - least deprived | 55 | | (21.74) | 95 | (18.96) | 0.327 |
|  | 2nd quintile | 43 | | (17.00) | 106 | (21.16) |  |
|  | 3rd quintile | 47 | | (18.58) | 99 | (19.76) |  |
|  | 4th quintile | 47 | | (18.58) | 92 | (18.36) |  |
|  | 5th quintile - most deprived | 54 | | (21.34) | 101 | (20.16) |  |
| **Block 2 – environ-mental** | **Living alone status** |  | |  |  |  |  |
|  | No | 192 | | (75.89) | 290 | (57.88) | <0.001 *** |
|  | Yes | 31 | | (12.25) | 100 | (19.96) |  |
|  | Unknown/no-3m | 30 | | (11.86) | 111 | (22.16) |  |
|  | **Social support (MOS-SSS)** |  | | |  | |  |
|  | Inadequate (MOS <80) | 91 | | (35.97) | 190 | (37.92) | 0.867 |
|  | Adequate (MOS >=80) | 161 | | (63.64) | 306 | (61.08) |  |
| **Block 3 - clinical** | **Tumour site** |  | |  |  |  |  |
|  | Colon | 147 | | (58.10) | 338 | (67.47) | 0.011 * |
|  | Rectum | 105 | | (41.50) | 161 | (32.14) |  |
|  | **Dukes’ stage** |  | |  |  |  |  |
|  | stage A | 41 | | (16.21) | 68 | (13.57) | 0.032 * |
|  | stage B | 118 | | (46.64) | 282 | (56.29) |  |
|  | stage C | 91 | | (35.97) | 142 | (28.34) |  |
|  | **Nodal status** |  | |  |  |  |  |
|  | N0 | 148 | | (58.50) | 327 | (65.27) | 0.032 * |
|  | N1-N2 | 90 | | (35.57) | 139 | (27.74) |  |
|  | **Any comorbidities** |  | |  |  |  |  |
|  | None | 86 | | (33.99) | 84 | (16.77) | <0.001 *** |
|  | Yes, non-limiting | 70 | | (27.67) | 142 | (28.34) |  |
|  | Yes, limiting | 52 | | (20.55) | 111 | (22.16) |  |
|  | Unknown/no-3m | 45 | | (17.79) | 164 | (32.73) |  |
| **Block 4 - treatment** | **Stoma** |  | |  |  |  |  |
|  | Yes | 108 | | (42.69) | 157 | (31.34) | 0.002 ** |
|  | No | 141 | | (55.73) | 337 | (67.27) |  |
|  | **Neoadjuvant therapy** |  | |  |  |  |  |
|  | None | 190 | | (75.10) | 413 | (82.44) | 0.020 * |
|  | Any (CT, RT, both) | 60 | | (23.72) | 84 | (16.77) |  |
|  | **Adjuvant therapy** |  | |  |  |  |  |
|  | None | 135 | | (53.36) | 353 | (70.46) | <0.001 *** |
|  | Any (CT, RT, both) | 117 | | (46.25) | 146 | (29.14) |  |
| **Block 5 - Personal factors** | **Self-efficacy (LORIG)** |  | | |  | |  |
|  | Low confidence | 27 | | (10.67) | 56 | (11.18) | 0.378 |
|  | Moderate confidence | 66 | | (26.09) | 102 | (20.36) |  |
|  | Confident | 102 | | (40.32) | 214 | (42.71) |  |
|  | Very confident | 54 | | (21.34) | 118 | (23.55) |  |
|  | **Clinical depression (CES-D)** |  | | |  | |  |
|  | No (<20 CES-D) | 188 | | (74.30) | 394 | (78.64) | 0.047 * |
|  | Yes (>=20 CES-D) | 64 | | (25.30) | 93 | (18.56) |  |
|  | **Clinical anxiety (STAI)** |  | | |  | |  |
|  | No (<40 STAI) | 132 | | (52.17) | 316 | (63.07) | 0.002 *** |
|  | Yes (>=40 STAI) | 118 | | (46.64) | 173 | (34.53) |  |

*Note*: missing values contributed less than 5% in each variable (not presented, but accounted for in the column percentages);

^1^ a Mann-Whitney test for continuous variables (all had a non-parametric distribution per age group confirmed by the Shapiro–Wilk and Shapiro–Francia tests) and a chi-squared test for categorical variables.

***p<0.001, **p<0.01, * p<0.05
