# Supplementary material for: Quality of life and health status in older adults (≥65 years) up to five years following colorectal cancer treatment: Findings from the ColoREctal Wellbeing (CREW) cohort study
Source: PLoS One. 2022 Jul 14;17(7):e0270033. doi: 10.1371/journal.pone.0270033 (PMC9282586; doi:10.1371/journal.pone.0270033)
Supplement: S3 Appendix — (DOCX) [file pone.0270033.s003.docx]

**S3 Appendix.** **The distribution of QLACS-GSS for older participants at each timepoint in CREW**

Note: * p<0.05, ** p<0.01, *** p<0.001; Mann-Whitney test was applied to identify statistically significant differences between baseline and each another timepoint.
