# Supplementary material for: Quality of life and health status in older adults (≥65 years) up to five years following colorectal cancer treatment: Findings from the ColoREctal Wellbeing (CREW) cohort study
Source: PLoS One. 2022 Jul 14;17(7):e0270033. doi: 10.1371/journal.pone.0270033 (PMC9282586; doi:10.1371/journal.pone.0270033)
Supplement: S4 Appendix — (DOCX) [file pone.0270033.s004.docx]

**S4 Appendix****. The distribution of older participants reporting any health problems by five EQ-5D domains at each timepoint in CREW**

Note: * p<0.05, ** p<0.01, *** p<0.001; Chi-squared test was applied to identify statistically significant differences between baseline and each another timepoint.
