# Supplementary material for: Quality of life and health status in older adults (≥65 years) up to five years following colorectal cancer treatment: Findings from the ColoREctal Wellbeing (CREW) cohort study
Source: PLoS One. 2022 Jul 14;17(7):e0270033. doi: 10.1371/journal.pone.0270033 (PMC9282586; doi:10.1371/journal.pone.0270033)
Supplement: Attachment — Submitted filename: Comments.doc [file pone.0270033.s005.doc]

Comments

This is a well-designed cohort study. But there is some minor disadvantages.

1. In the Abstract- Objectvie part, the author defined the age of the older participants to be greater than 75 years old, which is inconsistent with the whole manuscript.
2. In line 108, the logo *Insert Figure 1* should be delete.(in line 236, the logo*Insert Table 1* , and in line 277).
3. The statistical part should be carefully checked by a professional statistician.
4. In the Results-Participants part, it would be better to have a detailed screening flowchart.
5. In line 301, the author wrote “Adequate social support was predictive of less problems with usual activities (OR=0.62, CI95% 0.42 to 0.91) and anxiety/depression (OR=0.56 CI95% 0.37 to 0.84)”. The format in the brackets can be modified to (OR=0.62, 95%CI: 0.42- 0.91), and the author should carefully modify other expressions.
6. The author should double check the reference format. For example, the reference 09.
7. For the tables in the manuscript, is it more appropriate to use a three-line table?
8. In the table 1, could you give test statistics, such as t-value or chi-square value?
9. The figure 1 legends could deleted “(19)”. And the Figure 1. should be revised as “Figure 1”. The full name of “LQ” and ” UQ” should be given in the last note.
10. The title of each paragraph could delete the underline. For example, Abstract should revised as Abstract. AND Introduction ...
