# Supplementary material for: Quality of life and health status in older adults (≥65 years) up to five years following colorectal cancer treatment: Findings from the ColoREctal Wellbeing (CREW) cohort study
Source: PLoS One. 2022 Jul 14;17(7):e0270033. doi: 10.1371/journal.pone.0270033 (PMC9282586; doi:10.1371/journal.pone.0270033)
Supplement: Attachment — Submitted filename: Response to Reviewers.docx [file pone.0270033.s006.docx]

**Quality of life and health status in older adults (≥65 years) up to five years following colorectal cancer treatment: Findings from the ColoREctal Wellbeing (CREW) cohort study**

**Response to Reviewers**

We thank the reviewers for their helpful comments. Please find our responses in the table below.

Reviewer 1 Comments

| Reviewer Comment | Author Response |
| --- | --- |
| In the Abstract- Objective part, the author defined the age of the older participants to be greater than 75 years old, which is inconsistent with the whole manuscript. | Changed in manuscript  The sentence:  ‘Colorectal cancer (CRC) is common in older adults, with more than 40% of diagnoses in people aged ≥75 years.’  Has been replaced with the sentence:  ‘Colorectal cancer (CRC) is common in older adults, with more than 70% of diagnoses in people aged ≥65 years.’ |
| In line 108, the logo *Insert Figure 1* should be delete.(in line 236, the logo*Insert Table 1* , and in line 277). | ‘*Insert Figure 1*’ has been changed to ‘**Fig 1. Predictors for older people with CRC mapped onto Foster and Fenlon’s conceptual model of health and wellbeing recovery following cancer treatment’**  Tables are now included in the manuscript as per the PLOS One submission guidelines:  ‘*Insert Table 1*’ has been changed to ‘**Table 1. Distribution of baseline characteristics for total older CREW participants (≥65 years) and by four age sub-groups for older participants’** followed by Table 1 and some notes under the Table  ‘*Insert Table 2*’ has been changed to ‘**Table 2. Log-linear regression model predicting the QLACS-GSS score over the 5-year follow-up post-surgery, predictors collected at baseline (pre-surgery), presented coefficients converted to percentage change and 95% confidence intervals (CI)’** followed by Table 2 and some notes under the Table  ‘*Insert Table 3*’ has been changed to ‘**Table 3. Logistic regression models predicting health problems separately in five EQ-5D domains over the five-year follow-up post-surgery, predictors collected at baseline (pre-surgery), presented coefficients converted to odds ratios (ORs) and 95% confidence intervals (CI)’** followed by Table 3 and some notes under the Table |
| The statistical part should be carefully checked by a professional statistician. | This has been done |
| In the Results-Participants part, it would be better to have a detailed screening flowchart. | A screening flowchart has now been included as Fig 2 on page 11. However, we note that it does make the section longer. |
| In line 301, the author wrote “Adequate social support was predictive of less problems with usual activities (OR=0.62, CI95% 0.42 to 0.91) and anxiety/depression (OR=0.56 CI95% 0.37 to 0.84)”. The format in the brackets can be modified to (OR=0.62, 95%CI: 0.42- 0.91), and the author should carefully modify other expressions. | ‘CI95%’ has been changed to ‘95%CI:’ throughout |
| The author should double check the reference format. For example, the reference 09. | References checked, two (9 and 36) had details missing, they are now complete |
| For the tables in the manuscript, is it more appropriate to use a three-line table? | We think this is a question for the copy editor |
| In the table 1, could you give test statistics, such as t-value or chi-square value? | We feel adding another column to the table would make it too large and potentially more complicated |
| The figure 1 legends could deleted “(19)”. And the Figure 1. should be revised as “Figure 1”. | ‘19’ has been deleted and ‘Figure 1.’ has been changed to ‘Fig. 1’ as per the author instructions |
| The full name of “LQ” and ” UQ” should be given in the last note. | The full names have been added to the note |
| The title of each paragraph could delete the underline. For example, Abstract should revised as Abstract. AND Introduction…. | Level 1, 2 and 3 headings have been formatted as per the author guidelines |

Reviewer 2 Comments

| Reviewer Comment | Author Response |
| --- | --- |
| lots of data - is it possible to make it more succinct? Suggest making it easier to read for a surgical audience | The results section has been shortened  When referring to study timepoints in the results section, we have used ‘pre-surgery’, ‘post-surgery’ more often to increase relevance for surgical audience |
| Suggest bold and definitive conclusive statements to define your point for each section - at the moment is reads as a lot of information but I am not being guided as to what you are specifically trying to say in each paragraph, 'lost in the direction of the narrative' through the results and discussion a little. | Results changed to highlight important findings at the beginning of each section. |
| Clinical relevance vs statistical relevance of some covariates (i,e, at least one limiting comorbidity) - this is confusing for the reader, i.e. is a limiting comorbidity hypertension vs stroke for example. In results - would be good to see p-values for your OR(CI95%), for the key defining results (i note that its already on table under *, ** etc). | ‘Limiting comorbidities’ are defined as being comorbidities which the patient has reported as limiting their daily activities. Patients chose from a list of 12 physical and mental health conditions and indicated to what extent that condition limited their daily activities. More explanation has been provided in the text. |
